# Histopathologic insights into the mechanism of anti-non-Gal antibody-mediated pig cardiac xenograft rejection

**DOI:** 10.1111/xen.12050

**Published:** 2013-09-20

**Authors:** Guerard W Byrne, Agnes M Azimzadeh, Mohamed Ezzelarab, Henry D Tazelaar, Burcin Ekser, Richard N Pierson, Simon C Robson, David K C Cooper, Christopher G A McGregor

**Affiliations:** 1 Institute of Cardiovascular Science, University College LondonLondon, UK; 2 Department of Surgery, Mayo ClinicRochester, MN, USA; 3 Division of Cardiothoracic Surgery, University of Maryland School of Medicine and Baltimore VAMCBaltimore, MD, USA; 4 Department of Surgery, Thomas E. Starzl Transplantation Institute, University of PittsburghPittsburgh, PA, USA; 5 Department of Laboratory Medicine and Pathology, Mayo ClinicScottsdale, AZ, USA; 6 Department of Medicine, Beth Israel Deaconess Medical CenterBoston, MA, USA

**Keywords:** cardiac transplantation, coagulation, complement activation, Gal epitope, orthotopic transplantation, xenotransplantation

## Abstract

The histopathology of cardiac xenograft rejection has evolved over the last 20 yr with the development of new modalities for limiting antibody-mediated injury, advancing regimens for immune suppression, and an ever-widening variety of new donor genetics. These new technologies have helped us progress from what was once an overwhelming anti-Gal-mediated hyperacute rejection to a more protracted anti-Gal-mediated vascular rejection to what is now a more complex manifestation of non-Gal humoral rejection and coagulation dysregulation. This review summarizes the changing histopathology of Gal- and non-Gal-mediated cardiac xenograft rejection and discusses the contributions of immune-mediated injury, species-specific immune-independent factors, transplant and therapeutic procedures, and donor genetics to the overall mechanism(s) of cardiac xenograft rejection.

## Introduction

Deceased human organ donation rates do not meet the demand for clinical transplantation. Changes in donor definition and legislation have not substantially closed this gap in supply. Potential alternatives to cardiac allotransplantation include mechanical devices, regenerative medicine applications, and xenotransplantation. Genetic modifications of organ-source pigs in concert with evolving immunosuppressive strategies have resulted in significant progress in cardiac xenotransplantation. Cardiac xenograft survival in the heterotopic pig-to-primate model has increased from a few hours to a median survival of 3 months with individual survival beyond 8 months [1–3].

Major contributions to this progress have been recognition of (i) the importance of antibodies directed to pig galactose-α1,3-galactose (Gal) epitope in xenograft rejection [4–7]; (ii) the potential protective effects of human complement regulatory protein (hCRP) transgenes [8,9]; and (iii) the development of therapies to deplete [10–14] or block anti-Gal antibody in vivo [15–19], culminating in the genetic elimination of the Gal antigen from the donor pig (the α1,3-galactosyltransferase gene knockout [GTKO] pig) [20–22]. Xenotransplantation is now in an era of anti-non-Gal antibody-mediated rejection.

The authors, members of the NIAID-supported Consortium on Immunobiology of Xenotransplantation, have extensively reported on cardiac xenotransplantation. In this review, the group assesses the histopathology of anti-non-Gal antibody-mediated cardiac xenograft rejection and discusses the implications this may have for future research strategies.

## Early anti-non-Gal antibody-mediated rejection: first evidence of a new histopathology

**Fig. 1:**
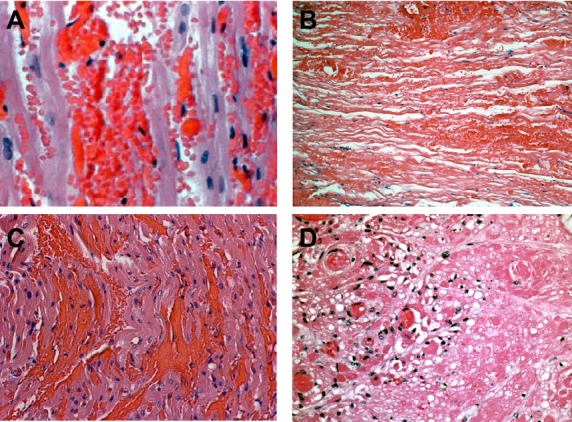
Histopathology of xenograft rejection. The figure shows a comparison between anti-Gal and non-Gal antibody-mediated cardiac xenograft rejection. All panels show hematoxylin and eosin staining. A. Anti-Gal antibody-induced hyperacute rejection of a Gal-positive heart showing widespread intravascular hemorrhage characteristic of HAR. B. Anti-Gal antibody-mediated delayed xenograft rejection (DXR) of a Gal-positive heart on post-operative day 10. The rejected graft shows vascular injury, hemorrhage, and coagulative necrosis characteristic of anti-Gal-mediated DXR. C. Non-Gal antibody-mediated hyperacute rejection of a GTKO heart 90 min after reperfusion showing intravascular hemorrhage similar to that seen in Gal-mediated HAR (panel A). D. Non-Gal-mediated DXR on post-operative day 92 of a Gal-positive CD46 transgenic heart showing thrombotic microangiopathy. The recipient in panel D received chronic alpha-Gal polymer infusions to block anti-Gal antibody. Original magnification A and C 400×, B and D 200× (Panel C adapted from: McGregor CGA, et al. Cardiac xenotransplantation: progress toward the clinic. Transplantation. 2004: 78: 1569–1575.)

The initial barrier to xenotransplantation was hyperacute rejection (HAR) caused by complement-mediated endothelial cell (EC) destruction directed by preformed anti-Gal antibody. The histopathology of HAR is predominantly characterized by rapid graft failure and widespread intravascular hemorrhage (Fig. 1A,C, Table 1). This is accompanied by vascular antibody, complement, and fibrin deposition with the formation of platelet-rich thrombi (not shown) [23–27]. Improved xenograft survival was not reliably achieved until methods were developed to block the effects of complement and anti-Gal antibody. Early attempts depleted anti-Gal antibody through pig-specific organ perfusion [10,23,24], plasmapheresis, or affinity immunoadsorption [11–14,28,29]. These studies demonstrated the dominant role of anti-Gal antibody in graft rejection [14,28–30], but provided only temporary antibody reduction. An induced anti-Gal antibody response led to delayed xenograft rejection (DXR) also characterized by interstitial hemorrhage, vascular antibody and complement deposition with diffuse platelet-rich fibrin thrombosis (Fig. 1B, Table 1). Unlike HAR, DXR occurs over the course of days to weeks, and vascular antibody and complement deposition, nearly universal in HAR, is more variable in DXR. This is due in part to the efficacy of different modalities (hCRP transgenic organs, cobra venom factor, plasmapheresis, or soluble complement inhibitors) used to limit antibody-dependent complement-mediated injury.

**Table 1:** Histology of cardiac xenograft rejection

Donor type	HAR	DXR^a^	TM/CC^a^,^b^
Wild type	• Acute rapid graft failure within minutes or hours after reperfusion	• Occurs days to weeks after transplantation	• Occurs days to weeks after transplantation
	• Extensive vascular antibody and complement deposition	• Vascular antibody and variable complement deposition	• Vascular antibody and complement deposition is variable
	• Prominent vascular injury and hemorrhage	• Intravascular injury and hemorrhage	• Minimal vascular hemorrhage
		• Prominent diffuse platelet-rich fibrin thrombosis	• Myocyte vacuolization.
	• Some platelet and fibrin thrombi may be present The expected outcome for transplantation of wild-type organs into untreated recipients	• Coagulative necrosis Requires pre-transplant therapies to limit immediate antibody- and complement-mediated graft injury	• Fibrin- and platelet-rich microvascular thrombosis.
			• Coagulative necrosis Requires rigorous pre- and post-transplant prevention of an anti-Gal antibody response
GTKO	Histology is comparable to wild-type donor organs, but the frequency of GTKO HAR is dramatically lower.	• Occurs days to months after transplantation.	
		• Vascular antibody and complement deposition is variable	
		• Minimal intravascular hemorrhage	
		• Myocyte vacuolization	
		• Fibrin- and platelet-rich microvascular thrombi	
		• Coagulative necrosisTypical histopathologic picture in GTKO organs in immune-suppressed recipients with low-to-moderate levels of anti-non-Gal antibody.	

a DXR and TM/CC typically show low levels of polymorphonuclear neutrophil and macrophage graft vascular adhesion and infiltration, with little apparent lymphocytic infiltrate. In TM/CC, increased levels of macrophage infiltration may accompany systemic innate cell activation.

b TM and CC may occur individually or in combination. TM is localized to the graft, and CC is an intravascular process with significant recipient thrombocytopenia and systemic fibrin consumption.

**Fig. 2:**
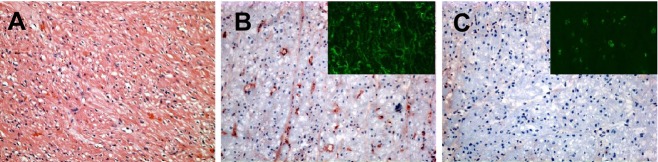
Anti-non-Gal antibody-mediated cardiac xenograft rejection. This figure shows the immunohistopathology of anti-non-Gal antibody-mediated DXR for Gal-positive CD46 pig heart protected from anti-Gal antibody by continuous infusion of an α-Gal polymer. A. Hematoxylin and eosin stain showing ischemic injury and myocardial coagulative necrosis in a graft with ongoing rejection at 113 days. B. Immunohistochemical staining of the same graft showing positive vascular IgM deposition. The insert shows immunofluorescence staining for fibrin. C. Negative immunohistochemical staining for C5b. The insert shows a low level of positive immunofluorescence staining for CD41 platelet thrombi. All photomicrographs at 200×. (Immunohistochemical staining in panels A–C adapted from: McGregor CGA, et al. Cardiac xenotransplantation: progress toward the clinic. Transplantation. 2004: 78: 1569–1575.)

Enduring reduction in anti-Gal antibody in vivo was successfully achieved using continuous or intermittent infusion of non-antigenic Gal polymers [1,19,27,31–34]. Of relevance to anti-non-Gal antibody-mediated GTKO pig xenograft rejection, these earlier studies are notable in that, for the first time, transplants using Gal polymers largely blocked the effects of both preformed and post-transplant-induced anti-Gal antibody, leading to a striking change in the histopathology of xenograft rejection [1,33,35]. Whereas anti-Gal-mediated DXR showed prominent interstitial hemorrhage (Fig. 1B), the histopathology of graft failure under conditions that efficiently blocked anti-Gal antibody was largely characterized by microvascular thrombosis with only focal evidence of interstitial hemorrhage (Fig. 1D, Table 1). This thrombotic microangiopathy (TM) was first explicitly noted by Houser using a poly-l-lysine Gal polymer and CD55 (hDAF) transgenic pig hearts [35]. The same histology was also reported using CD46 transgenic donor hearts and a polyethylene glycol Gal polymer [1,33,36,37] and in GTKO cardiac xenografts [3]. In the polymer studies, rejected cardiac xenografts uniformly showed vascular antibody deposition, fibrin, and platelet thrombi, with myocardial coagulative necrosis and ischemia (Fig. 2). Vascular complement deposition, variably measured by detection of C3, C4d, C5b, and C5b-9, was inconsistently observed and may be dependent on donor genetics. Lymphocytic infiltration of the graft was generally minimal or absent.

**Fig. 3:**
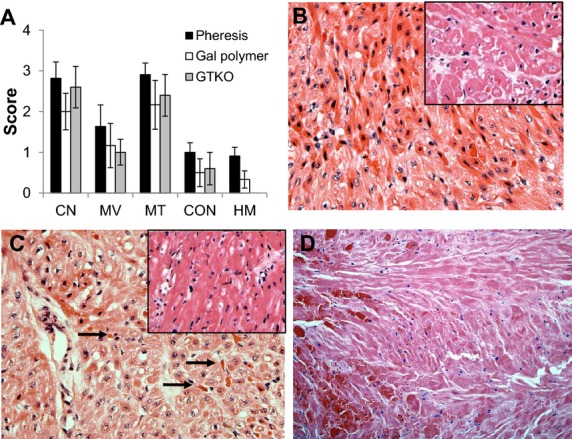
Histopathologic features of DXR in the absence of the effects of anti-Gal antibody. Data from three treatment groups are shown. (i) Recipient treated by plasmapheresis (Pheresis) to deplete anti-Gal antibody pre- and post-transplant; (ii) Chronic Gal-polymer-treated recipient to block anti-Gal antibody in vivo; and (iii) Transplantation of a GTKO donor heart. A. Histologic features of DXR in the absence of acute anti-Gal antibody. The intensity of major histopathologic features at explant (mean histology score ± standard error of the mean) are shown. (Abbreviations: CN, coagulative necrosis; MV, myocyte vacuolization; MT, microvascular thrombosis; CON, congestion; HM, hemorrhage.) B–D. Progressive development of DXR (H&E 400×). B. Cardiac biopsy from an apheresis-treated recipient (day 13 of 53) showing early (stage 1) DXR characterized by myocyte vacuolization with minimal microvascular thrombosis or systemic release of cardiac troponin. Insert shows a stage 1 biopsy (day 47 of 71) from a GTKO/CD55 heart (H&E 200×). C. Interim biopsy (day 15 of 21) of a heart from an apheresis-treated recipient showing progressive (stage 2) DXR, characterized by increased levels of microvascular thrombosis (arrows) and developing coagulative necrosis. Insert shows a stage 2 biopsy (day 14 of 26) of a GTKO/CD55 heart (H&E 200×). D. Representative histopathology of grafts at explant in all three groups (Portions of this figure adapted from data in Tazelaar HD, Byrne GW, McGregor CG. Comparison of Gal and non-Gal-mediated cardiac xenograft rejection. Transplantation. 2011: 91: 968–975).

This change in histopathology was attributed to sustained depletion of anti-Gal antibody. A recent histopathology comparison of cardiac xenografts under conditions where pre-transplant anti-Gal antibody was uniformly depleted and post-transplant induction of anti-Gal antibody was either partially muted by immunoapheresis, blocked by in vivo Gal polymers, or made irrelevant using GTKO donor hearts supports this conclusion [38]. Under these conditions, the major histopathologic features of developing and terminal xenograft rejection were the same for each group (Fig. 3A). Early evidence of rejection included vascular antibody deposition at 30 min after organ reperfusion and, at later time points, consistent myocyte vacuolization in the absence of appreciable microvascular thrombosis (Fig. 3B). As rejection progressed, based on the systemic release of cardiac troponin, diffuse microvascular thrombosis developed, eventually leading to myocardial coagulative necrosis and ischemic changes (Fig. 3C). At the time of graft failure, all three groups showed prominent microvascular thrombosis and coagulative necrosis with minimal interstitial hemorrhage or lymphocytic infiltration (Fig. 3D). Taken together, these results suggest that muting or elimination of the acute effects of preformed anti-Gal antibody reduced the intensity of humoral rejection, which likely limited the extent of interstitial hemorrhage. Gene expression analysis of these transplants suggested that a chronic state of antibody-mediated EC activation likely contributed to the development of TM [38].

## Histopathology of GTKO cardiac xenotransplantation

The initial study of heterotopic GTKO cardiac xenotransplantation reported no HAR and a median survival of 78 days [3]. This study was performed in recipients with minimal preformed anti-non-Gal antibody and used a well-established immunosuppressive regimen based on lymphocyte depletion, cobra venom factor (CVF), and chronic costimulation blockade. Recipients showed general hyporesponsive lymphocyte reactivity and had little evidence of an induced antibody response.

A detailed histology and immunohistology analysis was consistent with a progressive humoral rejection, resulting in widespread platelet-rich/fibrin-rich microvascular thrombi, myocardial ischemia, and necrosis, with focal interstitial hemorrhage [39]. Importantly, the degree of rejection was shown to be proportional to the level of vascular immunoglobulin and complement deposition, increased expression of recipient porcine tissue factor (pTF), formation of fibrin–platelet thrombi, and the frequency of EC apoptosis. Graft failure was also associated with a proportionate loss of CD39 expression. Cellular infiltration of the graft was minimal to mild and consisted mainly of monocytes with few lymphocytes.

These transplants showed that using GTKO organs effectively eliminated a role of anti-Gal antibody in graft rejection, but also clearly demonstrated the significance of anti-non-Gal antibody in the development of graft failure. Under these conditions, non-Gal-mediated GTKO heart rejection involved three major processes: (i) direct antibody-mediated EC injury, supported by the vascular deposition of antibody and terminal complement complexes in 7 of 8 grafts; (ii) EC activation, as evidenced by increased expression of pTF and vascular loss of CD39; and (iii) EC apoptosis that occurred relatively late in the rejection process. The development of these pathophysiologic processes progressed in parallel with histologic changes (microvascular thrombosis and coagulative necrosis), suggesting that TM within the graft resulted from the effects of immunoglobulin and complement, that is to say immune-mediated rejection.

However, other processes may also contribute significantly to graft thrombosis. These include systemic activation of recipient innate immune cells, leading to consumptive coagulopathy (CC) [40–42], as well as pig-specific deficiencies in the regulation of thrombosis [43–46]. Recent histologic analysis of GTKO graft failure is helping to identify when and how these processes may contribute to xenograft rejection.

## Early anti-non-Gal-induced immune injury

Cytotoxic anti-non-Gal antibody, with a titer typically 2- to 3-fold lower than anti-Gal antibody, is broadly present in human and non-human primate serum [47]. Despite this reduced titer, non-Gal antibody can in some instances have significant deleterious effects. A classic case of HAR has been reported in a GTKO heart [48,49]. Immediately post-transplant, the graft showed good contractilTableity. A 30-min biopsy showed normal myocardium, but with extensive vascular antibody deposition and moderate focal C5b deposition. By 90 min post-transplant, contractility had ceased and the histology showed widespread intramyocardial hemorrhage (Fig. 1C, Table 1) with extensive vascular antibody and complement deposition [see Fig. 2 in Reference [49]]. The timing, gross appearance, and histopathology of this graft were entirely consistent with an antibody-mediated HAR and did not differ from the histology of anti-Gal antibody-mediated HAR (Fig. 1A).

**Fig. 4:**
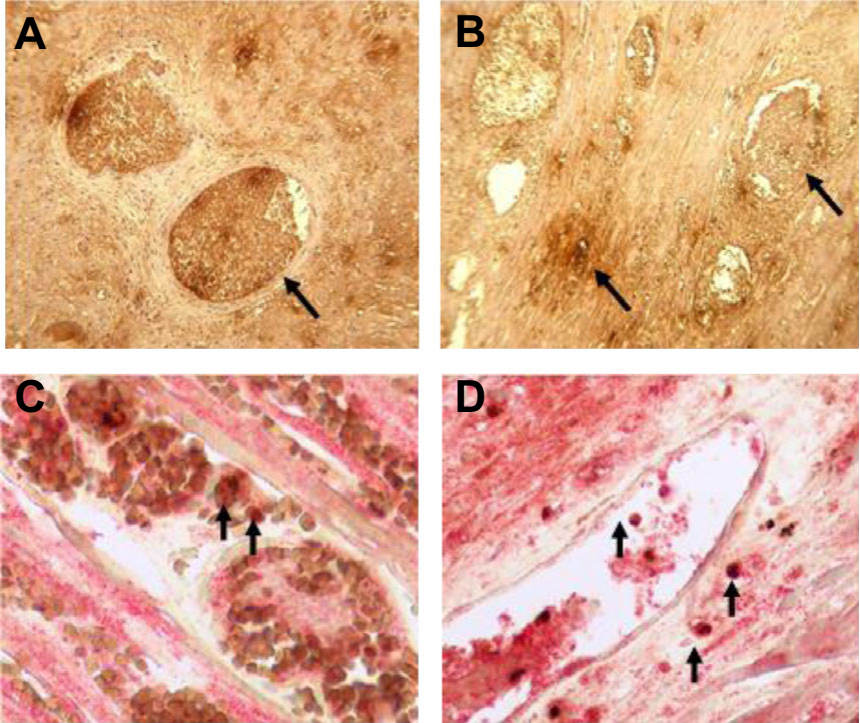
Expression of recipient TF in cardiac xenotransplantation. Immunohistochemical staining for recipient baboon tissue factor (bTF) expression in rejected cardiac xenografts. A. Staining for bTF in pig heart grafts that rejected at day 12. B. bTF expression at 8 weeks. Both photomicrographs in A and B show strong TF staining in thrombosed vessels and less staining in the interstitium (arrows) (×600). C and D. Colocalization of bTF (red stain) and macrophages (stained for CD68, brown) in heart grafts excised on day 12 (C) and at 8 weeks (D) is indicated by arrows (×600) (Reproduced with permission from Ezzelarab M, et al. Transplantation 2009; 87: 805–812).

α1,3-galactosyltransferase gene knockout pigs heart xenografts have also been reported to undergo early immune injury from preformed anti-non-Gal antibody, which did not result in HAR [34,41]. In these studies, GTKO graft survival was <1 day in the absence of immune suppression, but was extended to 2 to 12 days with “partial” immune suppression and up to 8 weeks with a “full” regimen. Xenograft rejection was complex, as the grafts, regardless of the efficiency of immune suppression, showed evidence of both humoral rejection, in the form of vascular antibody and complement deposition, and recipient innate immune cell activation. The innate cell activation was manifested as CC (defined by thrombocytopenia, low fibrinogen levels, prolongation of prothrombin and activated partial thromboplastin times, bleeding) and as a significantly increased level of intragraft neutrophil infiltration with a marked increase in recipient baboon tissue factor (bTF) expression from intragraft and graft adherent intravascular monocytes and macrophages (Fig. 4A–D, Table 1). Activation of recipient innate immune cells and induction of bTF expression have also been reported in kidney xenograft recipients subject to CC [42].

These results demonstrate that preformed anti-non-Gal antibody contributes to early graft injury, but also show clearly that under some conditions, systemic activation of the recipient innate immune cells can occur. This activation, resulting in increased recipient bTF expression on peripheral blood mononuclear cells, may be stimulated through antibody-mediated rejection or through cell–cell interactions that are independent of antibody-induced EC activation [42,50,51]. In both cases, activated recipient innate cells can exacerbate ongoing humoral injury or directly contribute to intragraft thrombosis.

## Coagulation and graft failure

The differential expression of TF either from circulating recipient monocytes or from donor graft endothelium (described above) is a key observation, which suggests that a combination of distinct mechanisms—local non-Gal antibody-mediated immune injury and systemic recipient innate cell activation—may contribute to overall intragraft thrombosis. What is clear is that similar heterotopic and orthotopic xenograft survival can be obtained with [3,35,41] and without [1,33,36,52,53] exogenous anti-coagulation and that to date, the thrombogenic process leading to terminal graft failure remains resistant to all forms of tested clinical anti-coagulation [3,36,37,52–54]. In the era of GTKO donor pigs, with the elimination of anti-Gal antibody-mediated rejection, the questions arise as to which of these mechanisms—systemic recipient monocyte activation or antibody-mediated intragraft EC activation—is the dominant stimulus for terminal intragraft thrombosis (rejection) and how best to prevent this process.

Systemic activation of recipient innate cells may lead to CC, as has been variably reported after pig-to-primate kidney, heart, and hematopoietic cellular xenotransplantation [30,40,55,56]. CC is not, however, universally observed [24,28,29,32,37,54,57,58] and therefore may not be a *de facto* inherent property of porcine organs. We suggest that a combination of four major factors—(i) antibody-mediated injury; (ii) pig-specific immune-independent factors; (iii) transplantation and certain transplant-related therapies; and (iv) donor porcine genetics—need to be considered as potential factors that contribute to recipient innate cell activation and its role in CC (Table 2).

**Table 2:** Factors that may influence the development of thrombotic microangiopathy in GTKO pig cardiac xenografts

1. Immune-mediated injury
Complement-mediated EC injury
Anti-non-Gal antibody-mediated EC activation
2. Pig-specific immune-independent factors
Porcine vWF, TFPI, and thrombomodulin incompatibility
Recipient platelet/monocyte and porcine EC interactions
3. Transplantation or treatment factors
Xenotransplant model: heterotopic vs. orthotopic
Complement targeting: cobra venom factor, C1 Inhibitor (C1INH)
Antibody depletion: plasmapheresis, extracorporeal immunoadsorption
Anti-CD154 mAb
Anti-coagulation: heparin, aspirin.
4. Donor genetics
Antigen reduction (GTKO, Neu5Gc-KO)
Human complement regulatory protein transgene expression (CD46, CD55, CD59)
Anti-coagulation and anti-thrombotic transgene expression (TBM, EPCR, CD39, TFPI)

### Antibody-mediated injury

The systemic release of anaphylatoxin C5a appears to be a key component of antibody-mediated injury, which may also secondarily promote systemic recipient innate cell activation. In xenotransplantation, C5a is most often produced through complement activation by antibody-mediated injury or as a consequence of CVF administration to deplete complement [59]. Elevated levels of C5a, reported in hemodialysis patients [60], suggest that plasmapheresis to remove anti-pig antibody may also increase circulating levels of C5a [10–14]. C5a is a potent inflammatory peptide that bridges the complement and coagulation cascades [61], promotes thrombosis [62,63], and contributes to innate and adaptive immune responses [64]. Antibody-mediated release of C5a within the graft can induce TF expression in pig ECs, recruit neutrophils and monocytes to the xenograft, induce TF expression on recipient neutrophils, and sensitize monocytes to express inflammatory cytokines [65]. The systemic release of C5a by CVF, while not sufficient to induce frank CC, may activate recipient neutrophils and monocytes and potentiate their response to inflammatory mediators produced from humoral injury to the graft. C5a release may contribute to the frequency of graft-infiltrating neutrophils and expression of bTF observed in hearts showing a mixture of humoral rejection and recipient innate cell activation.

### Pig-specific immune-independent factors

There are also pig-specific immune-independent factors that may contribute to development of CC. These include well-known incompatibilities involving porcine von Willebrand factor (vWF) and thrombomodulin (TBM), which have been recently reviewed [66]. Porcine vWF binds to human GpIb on platelets to cause a shear-independent aggregation. Because of this unusual cross-species interaction, accumulation of porcine vWF in the circulation of xenograft recipients over time would be anticipated to increase the tendency for systemic coagulation [67]. Likewise, the inefficient graft-specific production of activated protein C by porcine TBM may predispose the xenograft to intragraft thrombosis [68].

Cross-species cellular interactions between porcine ECs and resting human platelets and monocytes may also lead to systemic platelet activation and EC apoptosis [50,69]. Co incubation of porcine EC with resting human platelets or monocytes leads to platelet activation and platelet and monocyte expression of human TF. Activated platelets in turn express CD154 and can induce a CD154-dependent activation of resting porcine ECs. In vitro studies show that porcine EC can also be activated by binding of human polymorphonuclear neutrophils (PMNs) and natural killer (NK) cells [70,71]. PMN binding to pig ECs under flow conditions leads to intracellular calcium spikes and EC activation not seen in allogeneic EC [72]. This interaction results in a PMN respiratory burst, increased inflammatory gene expression, and elevated cytokine expression [73]. These changes may further recruit PMNs, monocytes, and leukocytes to the graft; increase transendothelial leukocyte migration; enhance monocyte and PMN TF expression; exacerbate intragraft inflammation and thrombosis; and increase EC sensitivity to NK cytotoxicity [70,74]. These cellular interactions occur independent of xenoreactive antibody and could, in principle, create an amplification cycle promoting CC by increasing systemic activation of recipient platelets and monocytes and intragraft activation of vascular ECs. It remains to be determined to what extent these in vitro cellular effects contribute to cardiac xenograft rejection.

Anti-CD154 mAb has been used as a chronic immunosuppressive agent in several pig-to-primate organ and cellular xenotransplantation models. Combined with other therapies, anti-CD154 mAb efficiently blocks induction of anti-pig antibody [3,31,35,41,42,75]. Use of an anti-CD154 mAb therapy has often been associated with the development of CC [3,31,34,41,55], although not with every anti-CD154 mAb-based regimen [76,77]. One hypothesis suggests that activated platelets, with CD154 surface expression, may be cross-linked by anti-CD154 mAb to promote thrombosis [78]. Whether this mechanism is active in allotransplantation remains under investigation [79]; however in xenotransplantation, platelet activation through cross-species non-immune cellular interactions such as those discussed above and chronic administration of anti-CD154 may increase the risk of CC. Taken together, severe perioperative platelet loss, a common sign of CC, could be due to insufficient immunosuppression (strong immune-mediated effects), intense non-immune cross-species cellular interactions, or milder cellular interactions exacerbated by reagents such as CVF and anti-CD154 mAb. Paradoxically, under some conditions, anti-CD154 mAb may also block CD154-dependent platelet adhesion to graft ECs and thereby inhibit EC activation [69].

### Transplantation and treatment factors

The transplantation model—heterotopic or orthotopic—may affect the development of CC, which has been primarily associated with heterotopic cardiac xenotransplantation [31,35,41,55]. In some instances, this may be due to sluggish blood flow and intrachamber thrombus formation detected by echocardiography in heterotopic transplants. Conceivably, soluble products derived from coagulation within the chambers of the heart may enhance both systemic coagulopathy and microvascular thrombosis within the xenograft. This process would likely not account for early perioperative thrombocytopenia.

In contrast, CC has generally not been reported after orthotopic cardiac xenotransplantation in the context of conventional immunosuppression [58,80–87] despite the requirement for cardiopulmonary bypass, which is known to activate platelets. The general absence of post-operative CC in orthotopic xenotransplantation may be affected by the need for full anti-coagulation at the time of the transplant and/or the higher flow rate of blood through the graft throughout the post-operative course.

### Donor genetics

An increasing variety of genetically modified pigs are becoming available (Table 3), which may help to minimize systemic coagulation and prolong graft survival. The most immediately accessible are GTKO donor pigs expressing hCRP transgenes. Transgenic expression of an hCRP creates an enhanced intrinsic barrier to complement activation and would be expected to minimize production of C5a. This may help to inhibit both antibody-mediated injury to the graft and systemic recipient innate cell activation. A recent comparison of heterotopic cardiac xenografts using GTKO and GTKO/CD55 hearts under carefully matched immune suppression showed a reduced frequency of C5b deposition in GTKO/CD55 hearts consistent with improved complement restriction in the graft [49]. Expression of CD55 appeared to prevent early graft injury as hyperacute rejection of a GTKO heart was observed in this study. Similar protection from early graft failure has been reported using other hCRPs [88 and A.M. Azimzadeh, unpublished observation]. In this matched study, expression of CD55 did not, however, lead to improved overall graft survival (GTKO median survival 21 days, range 0 to 128 days, and GTKO/hCD55 median survival 28 days, range 15 to 54 days). This may be due to chronic EC activation, as elicited non-Gal antibody can lead to complement-independent EC activation, which is not affected by hCRP expression [51]. Intragraft gene expression studies were consistent with the interpretation that graft rejection occurred resultant to chronic EC activation [49].

**Table 3:** Genetically modified pigs currently available for xenotransplantation research^a^

Gal antigen deletion or “masking”
α 1,3-galactosyltransferase gene knockout (GTKO)
Human H-transferase gene expression (expression of blood type O antigen)
Endo-β-galactosidase C (reduction in Gal antigen expression)
Human N-acetylglucosaminyltransferase III gene expression (GnT-III)
cytidine monophosphate-N-acetylneuraminic acid hydroxylase (Neu5Gc-KO)
Complement regulation by human complement regulatory gene expression
CD46 (membrane cofactor protein)
CD55 (decay-accelerating factor)
CD59 (protectin or membrane inhibitor of reactive lysis)
Anti-coagulation, anti-thrombotic, anti-inflammatory, and apoptosis gene expression or deletion
human tissue factor pathway inhibitor (TFPI)
human thrombomodulin (TBM)
human endothelial protein C receptor (EPCR)
human CD39 (ectonucleoside triphosphate diphosphohydrolase-1)
porcine von Willebrand factor deficiency (vWF natural mutant)
human A20 (tumor necrosis factor-alpha-induced protein 3)
human HO-1 (heme oxygenase-1)
human TNFRI-Fc (tumor necrosis factor-alpha receptor 1-Fc)
Suppression of cellular immune response by gene expression or down-regulation
porcine cytotoxic T-lymphocyte antigen 4 expression (CTLA4-Ig)
human modified CTLA4-Ig expression (LEA29Y)
CIITA-DN expression (swine leukocyte class II knockdown)
human TRAIL (tumor necrosis factor-alpha-related apoptosis-induced ligand)
human HLA-E β2-microglobulin expression (inhibits human natural killer cells cytotoxicity)
human CD47 (regulates species-specific CD47-dependent macrophage interactions)
Human FAS ligand expression (CD95L)

a Modified from Ekser B et al. [Ref [86]].

Pigs with combinations of genetic modification, for example GTKO with added transgenes, are available.

Cardiac xenotransplantation has also been reported using GTKO pigs expressing human CD46 [2]. In this study, immune suppression consisted of a unique combination of anti-thymocyte globulin, anti-CD20 mAb, anti-CD154 mAb, and CVF induction therapy, with mycophenolate mofetil and anti-CD154 mAb maintenance therapy, with daily aspirin and continuous heparin infusion post-transplant. With “full immune suppression”, median graft survival was 71 days, but with “partial immune suppression” (anti-CD20 mAb withheld), median graft survival decreased to 8 days, and histopathology showed evidence of rejection. The effectiveness of human CD46 expression to prevent early complement-mediated injury was unclear in this study and may have been confounded by early CC.

Pigs expressing human TBM have been reported ([89,90] and (Ayares D. Personal Communications)). Expression of human TBM appears to correct the molecular incompatibility between pigs and primates and may significantly improve thromboregulation within the graft. Additional new genetics (Table 3) may further improve thromboregulation (e.g., tissue factor pathway inhibitor [TFPI], endothelial protein C receptor [EPCR], CD39) or reduce inflammation (HO-1, TNFRI-Fc, A20, CD39) and ischemia–reperfusion injury (CD39, HO-1) [91,92]. Expression of these genes may act to limit recipient innate cell activation and reduce the tendency toward CC.

The potential effects of common therapies, including plasmapheresis, CVF, and anti-CD154 mAb, discussed above, are individually not sufficient to induce CC. Rather, it appears to be the combination of therapies, donor genetics, and transplant model that may predispose toward the development of CC. In future, a focus on the orthotopic transplant model, elimination of CVF therapy, or achieving complement inhibition with newer complement agents such as eculizumab [93,94]; substitution of anti-CD154 mAb by other biologic agents; and the use of GTKO donors with intrinsic complement regulation and genetically modified to reduce the pig-specific immune-independent factors (e.g., that contribute to coagulation dysregulation) should minimize the tendency for recipient innate cell activation and CC and may further diminish graft failure from TM.

## Orthotopic cardiac xenotransplantation

Most cardiac xenotransplantation studies have used the abdominal heterotopic model where the main focus has been to extend xenograft survival by developing an optimal combination of donor genetics and immune suppression. This has been a successful approach as median heterotopic graft survival has increased to 3 months, cellular rejection is minimal, and the induction of anti-non-Gal antibody is generally blocked [1–3]. Replication of these results in life-supporting transplants would show the efficacy of cardiac xenotransplantation and support limited clinical testing [95]. Orthotopic cardiac xenotransplantation is a more technically challenging model, and both the number of groups who have performed these transplants and the number of transplants are limited [58,80–86].

**Fig. 5:**
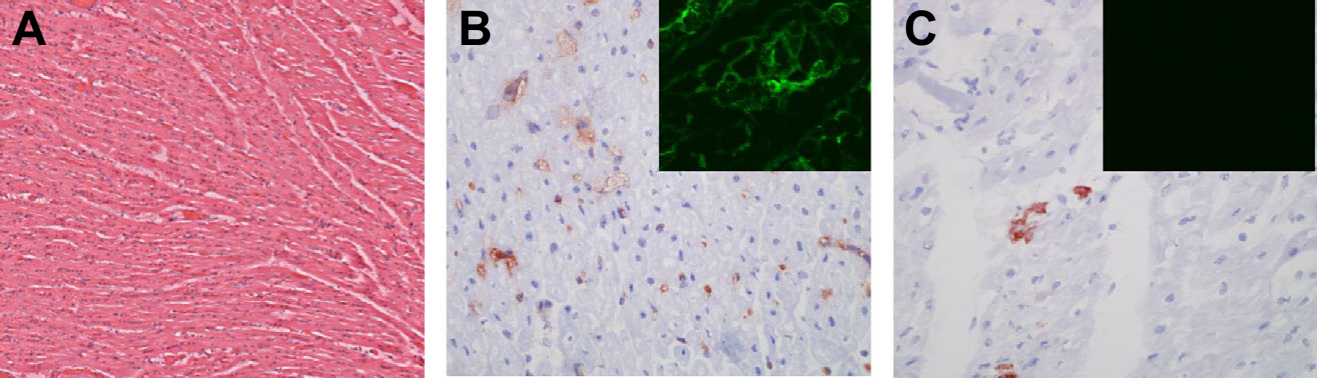
Histology of orthotopic cardiac xenografts. A. Hematoxylin and eosin stain of heart explanted after 57 days. B and C Immunohistology on day 57 for vascular IgM (B) and C5b (C). Insert in B shows moderate fibrin staining. Insert in C shows minimal to negative staining for CD41 platelet thrombi.

Overall reported orthotopic graft survival ranges from <1 to 57 days, but with only 9 of 54 reported grafts surviving for >2 weeks [87]. Recipient death most often occurs either within the first 48 h due to perioperative cardiac xenograft dysfunction (PCXD) or, in recipients that survive beyond 48 h, from post-operative complications. Early orthotopic transplants using CD55 transgenic organs without specific therapy to block anti-Gal antibody had graft survival of 5–39 days [80,81]. The histopathology of organ rejection included intense vascular IgM and complement deposition, thrombosis, and interstitial hemorrhage, which was consistent with anti-Gal antibody-induced DXR [96,97]. Subsequently, Gal polymer therapy was used to block anti-Gal antibody in recipients of CD55 transgenic grafts [84,85]. In this case, rejection correlated with the induction of anti-non-Gal antibody, and the histology was consistent with TM, including antibody and complement deposition, fibrin thrombosis, and myocyte necrosis. In studies using Gal-positive CD46 hearts with Gal polymer therapy, or GTKO/CD55 hearts, graft survival ranged from 14 to 57 days without apparent rejection [86,98], but with complications associated with immunosuppressive drug therapy. Explanted hearts exhibited vascular antibody binding with minimal complement deposition. There were variable levels of fibrin deposition and little evidence of CD41-stained platelet thrombi (Fig. 5A–C). Histologic injury, characterized by myocyte necrosis, varied from minimal to mild in 4 of 5 recipients.

All studies involving orthotopic cardiac xenotransplantation have reported a high frequency of PCXD in which 40–60% of transplants failed within the first 48 h [87]. A similar failure rate is not observed after abdominal heterotopic cardiac xenotransplantation, where post-operative graft function, based on contractility, is generally satisfactory. The etiology of PCXD is unknown, but its prevalence after orthotopic xenotransplantation suggests that it results from the physiologic demands placed on the graft. In our experience, using Gal polymer and CD46 hearts or GTKO:CD55 donor hearts, the diagnosis and histopathology of PCXD-affected heart (absent clear iatrogenic events) are distinct from HAR or DXR. PCXD hearts weaned from cardiopulmonary bypass are reliant on continuous and often high levels of inotropic support. Repeated attempts to wean these hearts from inotropes are unsuccessful and lead to a progressive negative cycle of deteriorating cardiac function. The histology of PCXD-affected hearts (see Fig. 1C in Reference [98]) shows vascular antibody deposition with occasional diffuse fibrin deposits. There is however minimal platelet thrombi, vascular congestion, complement deposition, or thrombosis [58,80–86]. It seems likely that severity of PCXD is affected by heightened porcine sensitivity to ischemia–reperfusion injury and the effects of acute anti-non-Gal antibody-mediated injury.

In the absence of rejection, life-supporting cardiac xenografts explanted between 9 and 57 days universally exhibit vascular antibody deposition (Fig. 5B). Generally, these grafts showed minimal or focal histologic evidence of rejection and were largely free of platelet thrombi (insert Fig. 5C). In an unbiased genomewide analysis of porcine gene expression, both (i) grafts explanted due to PCXD; and (ii) surviving xenografts recovered at recipient death, but without frank rejection, exhibited an array of changes in cardiac gene expression indicative of inflammation and myocardial injury [98]. Some of these same changes in gene expression have also been noted in rejected heterotopic cardiac xenografts [99]. These observations indicate that despite life-supporting cardiac function and well-preserved myocardial histology, surviving orthotopic xenografts remain under continuous adverse stimulation, likely due to immune-mediated injury. The altered pattern of gene expression detected in these explanted hearts is compelling evidence of protracted EC activation. This suggests that in the presence of vascular antibody deposition, the general absence of microvascular thrombosis is maintained by a dynamic hemostatic regulatory response, which counters the thrombotic effects of antibody-induced EC activation. Further understanding of this dynamic process will likely open new opportunities for therapeutic and genetic interventions, which affect the balance of thrombosis and fibrinolysis.

## Future strategies

Future strategies in xenotransplantation research should be directed toward improving graft survival, thereby enhancing the efficacy of this potential clinical therapy. For cardiac xenotransplantation, we believe that this goal can be reached by focusing on three main issues.

First, for clinical orthotopic cardiac xenotransplantation, it is essential to limit the frequency of fatal PCXD. PCXD appears to be a multifactorial phenomenon related to anti-non-Gal antibody-induced immune injury and pig-specific sensitivities, such as the effects of transplantation trauma, cardiopulmonary bypass, ischemia–reperfusion injury, and vasospasm. Reducing the frequency of PCXD will likely require the development of improved methods of organ preservation and early therapeutic interventions to limit acute post-operative immune injury. For example, cariporide, a type I sodium–hydrogen exchange inhibitor, has been shown to limit ischemia–reperfusion injury in a porcine cardiac transplant model and improve organ preservation [100,101]. This or a similar pharmaceutical approach to improve organ preservation coupled with the transplantation of GTKO hearts expressing one or more hCRPs with possible antibody depletion prior to transplant may be effective to alleviate early antibody-mediated graft injury and reduce the frequency of fatal PCXD. Prophylactic use of anti-cytokine reagents, such as etanercept (Enbrel) to block TNF-α [102,103] or anakinra (Kineret) [104] to block IL-1 may be used to limit perioperative inflammation of vascular endothelial cells [105] and may further reduce the potential for PCXD. Donor hearts expressing elevated levels of CD39 are resistant to ischemia–reperfusion injury and may represent a non-pharmaceutical solution to modulate PCXD [106].

Second, the prevalence of vascular antibody and complement deposition in rejected GTKO cardiac xenografts, even in the apparent absence of an induced antibody response, indicates that a better understanding of the nature of anti-non-Gal antibody and further optimization of immune suppression would be valuable. In non-human primates, anti-pig non-Gal antibody responses to porcine EC proteins involved in autoimmunity, thrombosis, inflammation, and complement regulation (CD9, CD59, CD46, EPCR, Annexin 2A) [48] and an induced anti-glycan response directed to a carbohydrate encoded by the porcine glycosyltransferase, α1,4-N-acetylgalactosaminyltransferase have been observed [107,108]. In human sera, the induced anti-non-Gal antibody response to porcine proteins is not as well defined [109]. Humans, however, have preformed antibody to N-glycolylneuraminic acid (Neu5Gc), which is a unique anti-glycan response not present in non-human primates [110–113]. Anti-Neu5Gc antibody is estimated to constitute 7–13% of the preformed anti-pig non-Gal human antibody repertoire [114], and an induced anti-Neu5Gc antibody response has been detected after clinical porcine islet transplantation [115]. The potential for anti-Neu5Gc antibody to contribute to cardiac xenograft rejection remains unclear because anti-Neu5Gc antibodies are not present in non-human primates and therefore do not contribute to histopathology in experimental pig grafts [116–118]. Recently, pigs with a targeted mutation in cytidine monophosphate-N-acetylneuraminic acid hydroxylase (CMAH) have been produced on the GTKO background [119]. These double knockout pigs, which lack expression of Gal and Neu5Gc, are a promising development for clinical xenotransplantation, which remains to be tested.

The relative contribution of anti-glycan compared with anti-protein antibody to xenograft rejection as well as the pathogenicity of any individual non-Gal specificity is not clear. This is an important point, as additional genetic modification to eliminate or substitute common porcine protein target antigens is probably not feasible, but elimination of porcine expression of other glycans (in addition to Gal) is. The pathogenicity however of antibodies to other glycans, other than Gal, has not yet been shown [120–122].

Little is known about the origin of non-Gal antibodies bound to the vasculature of cardiac xenografts. Are these germ-line-encoded antibodies, cross-reactive affinity-matured antibody derived from an unrelated immune challenge, or the product of an induced xenograft-specific immune response? Depletion of mature B cells perioperatively has been associated with better graft outcome and reduced humoral sensitization [1,2], but this approach is not expected to affect existing plasma cells. Additional new therapeutic modalities, such as the proteasome inhibitor, bortezomib [123], may be useful to deplete plasma cells, and existing immune suppression regimens may be modified to incorporate B-cell-specific therapeutics, such as lymphoblast-B (belimumab) [124,125], to further limit any weak induction of anti-non-Gal antibody.

Third, it is essential to evaluate the contribution that new pigs expressing anti-coagulant transgene function will have on xenograft rejection, thrombosis, and systemic coagulopathy. Transgenic pigs expressing human TBM, EPCR, or CD39 have been produced [90,106,126–129]. Expression of human TBM appears to correct the primary porcine EC deficiency in activated protein C production [126]. Improved thrombin regulation may also reduce the T-cell-dependent adaptive immune response as thrombin-activated porcine aortic ECs induce increased human T-cell proliferation [130].

Expression of CD39 in conjunction with CD73 leads to local production of adenosine, which is a potent inhibitor of thrombosis. Transgenic mice expressing human CD39, although fully viable, show an increased capacity to produce adenosine, have impaired platelet aggregation, and prolonged bleeding times. In a mouse cardiac transplant model, these mouse hearts are protected from thrombosis [131]. In a warm renal ischemia model, resistance to ischemia–reperfusion injury has been demonstrated [131,132]. Hearts from transgenic pigs expressing human CD39 similarly showed a reduction in infarct size after coronary occlusion and reperfusion [106].

Initial experience with heterotopic heart transplantation in the hCD39, hCRP, and hTBM multitransgene GTKO pig-to-baboon model indicates that in the presence of CVF and anti-CD154 mAb therapy, high level expression of hCD39 by the porcine vasculature is associated with less thrombocytopenia and better maintenance of plasma fibrinogen levels in recipient baboons (B. Ekser, unpublished observation).

These data suggest that high level expression of hCD39 on this genetic background with concurrent validated expression of hCRP may have two benefits: first to prevent ischemia–reperfusion injury that might help prevent PCXD and second as an anti-thrombotic to prevent intragraft thrombosis. Further testing these and other new genetically modified donor swine will be required in both heterotopic and orthotopic cardiac transplant models to gauge the impact of these genes on PCXD, intragraft thrombosis, and systemic coagulation parameters in recipient baboons. In any case, CVF should be avoided as GTKO pigs expressing hCRPs are now available, and CVF may exacerbate recipient innate cell activation.

## Conclusions

Cardiac xenograft survival has improved significantly since the introduction in the early 1990s of the first transgenic pigs in which a hCRP was expressed [8,9]. GTKO pig organs have eliminated the need for specific anti-Gal antibody therapy, and multiple new transgenic modalities are being developed to regulate both the immune response and coagulation activation. Drugs with greater specificity toward B cells and antibody-producing plasma cells have been approved, which may improve immune regulation. PCXD has been identified as a hurdle, and aggressive investigation of this phenomenon is necessary. As new genetic and pharmacologic technologies to improve porcine cardiac preservation, control humoral and cellular immune responses, and limit thrombosis and inflammation are tested, we anticipate further significant improvements in cardiac xenograft survival, which will support clinical application.

## Abbreviations

bTF: baboon tissue factor
CC: consumptive coagulopathy
CVF: cobra venom factor
DXR: delayed xenograft rejection
EC: endothelial cell
Gal: galactose-α1,3-galactose
GTKO: α1,3-galactosyltransferase gene knockout pigs
HAR: hyperacute rejection
hCRP: human complement regulatory protein
mAb: monoclonal antibody
Neu5GC: N-glycolylneuraminic acid
PCXD: perioperative cardiac xenograft dysfunction
pTF: porcine tissue factor
TBM: thrombomodulin
TF: tissue factor
TM: thrombotic microangiopathy
vWF: von Willebrand factor
